# Bacterial Antimicrobial Resistance in Meat Products—Current Concepts

**DOI:** 10.3390/foods14162792

**Published:** 2025-08-11

**Authors:** Vladimir S. Kurćubić, Matija D. Munjić, Marko P. Dmitrić, Saša Živković, Slaviša B. Stajić, Igor Tomasevic

**Affiliations:** 1 Department of Food Technology, Faculty of Agronomy, University of Kragujevac, Cara Dušana 34, 32000 Čačak, Serbia; matija.munjic@kg.ac.rs; 2 Veterinary Specialist Institute Kraljevo, Žička 34, 36000 Kraljevo, Serbia; markodmitric@gmail.com (M.P.D.); sasha98@live.se (S.Ž.); 3 Department of Animal Source Food Technology, Faculty of Agriculture, University of Belgrade, Nemanjina 6, 11080 Belgrade, Serbia; stajic@agrif.bg.ac.rs; 4 DIL German Institute of Food Technology, Prof.-von-Klitzing-Str. 7, D-49610 Quakenbrück, Germany

**Keywords:** antimicrobial resistance (AMR), food chain, meat, meat processing, meat

## Abstract

This paper presents the main trends in antimicrobial resistance (AMR), as well as their health and other consequences; current knowledge about the emergence, spread, and mechanisms of AMR; and the progress to date in understanding possible pathways of resistance through the food chain and the role of food as a vector of antibiotic-resistant bacteria. We have reviewed the main approaches to the prevention and control of the development, selection, and spread of AMR in food-producing animals (FPAs) and the meat industry; bacterial AMR in FPA; the most significant and dangerous pathogens that show AMR; transmitted by meat and meat products; strategies to prevent the occurrence of AMR microorganism infections; and, recently, AMR monitoring and surveillance programs in meat production and processing. This study reviews the results of various studies, as well as inspiring and motivating reviews, that address the state of the art of AMR in targeted diverse niches that are integrated by the multidisciplinary “One Health” approach, as well as future strategies for reducing AMR. To successfully address the challenges associated with AMR, it is necessary to integrate monitoring and surveillance across the environment–raw materials–food (meat)–people continuum. It is necessary to permanently improve and expand the NCBI Pathogen Detection Isolate Browser (NPDIB) database and supplement it with the results of future research to identify and investigate genes that coordinate stress response and AMR.

## 1. Introduction

The use of chemical methods for controlling microorganisms in food carries with it a number of risks, such as drug resistance, chemical residues, and environmental pollution [1,2]. Global mapping and assessment of the importance of antibiotic consumption for the treatment of diseased animals in the future was pioneered and published by van Boeckel et al. [1]. Globally, one of the most current and significant challenges today when it comes to human and food-producing-animal health is the emergence of systemic and multidisciplinary antimicrobial resistance (AMR). Lobanovska and Pila [3] report that AMR was discovered as early as the 1940s, shortly after Fleming’s discovery of penicillin (1928), and that strains of *Escherichia coli* (*E. coli*) can produce penicillinase, which can nullify the therapeutic effects of penicillin. Lakhundi and Zhang [4] described that somewhat later, penicillin resistance was also confirmed in strains of *Staphylococcus aureus* (*S. aureus*) and *Streptococcus pneumoniae* (*S. pneumoniae*). Methicillin-resistant *S. aureus* (MRSA) produces a number of virulence factors and is considered a “SUPERBUG”. MRSA has the ability to create new strains (increased virulence and colonization capacity) by optimizing its content and gene expression [5,6]. The number of people who often consume antibiotics unnecessarily has increased dramatically, which unfortunately contributes to the development of AMR and progressively reduces the effectiveness of these important antimicrobial (AM) drugs [6].

**Figure 1:**
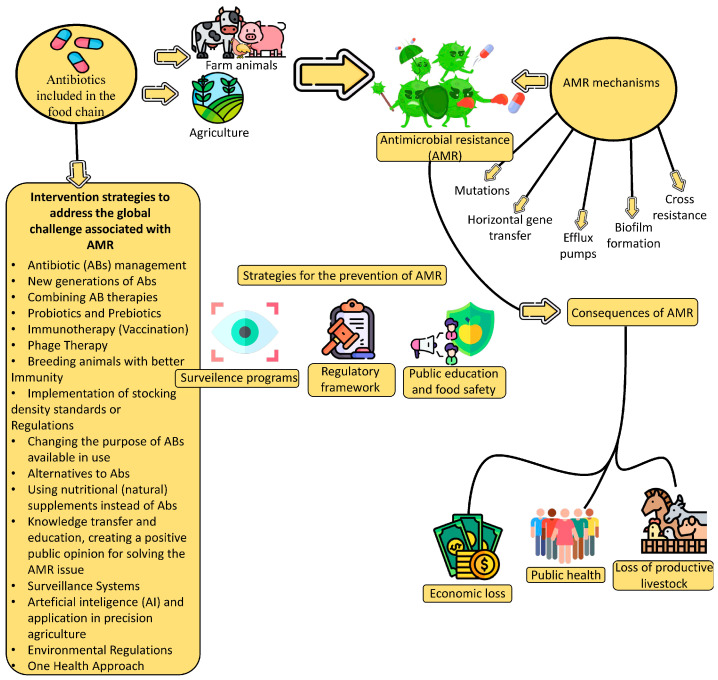
Emergence of AMR consequences and prevention strategies.

The use of antibiotics and other AM agents in a number of related ecological niches (agriculture, food-producing animals, and humans) leads to the emergence of resistant bacteria [7]. The widespread and often uncontrolled use of antibiotics in livestock is causing the development of antibiotic resistance in bacteria, with consequences for human and wildlife health. Restrictions on the use of antibiotics for livestock protection have been included in regulations to prevent drug resistance from evolving, and alternatives include pre- and probiotics, vaccines, and essential oils, which may be more cost-effective (Figure 1).

The major risk today arises from the high prevalence of non-pathogenic microorganisms that can transfer AMR genes to other microbial species, including foodborne pathogens (which are not frequent in foods, generally). Genes encoding resistance are transmissible between different bacteria in food-producing animals (FPAs) and also from them to bacteria in foods and in humans by horizontal gene transfer (i.e., transformation, conjugation, and transduction) [8]. The origin of drug resistance cannot be limited exclusively to primary production, as the food chain can itself generate resistance. Pressure exerted by the widespread use of biocides in food production (disinfectants, preservatives, and other chemicals or environmental and process conditions applied throughout the food distribution chain) has been shown to drive the adaptation of microbial populations to escape selective immune pressure by developing transient resistance [9,10].

According to the degree of resistance and modern nomenclature, bacterial resistance is designated as multidrug resistance (MDR), extensive drug resistance (XDR), pan-drug resistance (PDR), and difficult-to-treat resistance (DTR), depending on the number of different classes of antibiotics whose in vitro AM activity is proven [11,12,13,14,15,16,17,18,19]. In veterinary medicine, there are currently no standardized definitions for MDR, although numerous reports have been published on the potential for AMR to be on the rise among clinically important bacteria in livestock and companion animals [20,21], and there are attempts to apply the already-standardized definitions from human medicine for MDR, XDR, and PDR to animal pathogens and veterinary AM agents. These define MDR as an isolate that is not susceptible to at least one agent in at least three AM classes; XDR is an isolate that is not susceptible to at least one agent in all but one or two available classes; PDR is an isolate that is not susceptible to all agents in all available classes [12].

The potential risks of AMR expressed in zoonotic MDR pathogens that can lead to severe, often fatal diseases are raising concerns about the safe operation of the food industry, loss of consumer confidence, and reduced sales of animal-based food, causing economic losses [22]. Risk factors along the food chain (animal-based food as a carrier of bacterial-specific antibiotic resistance, including possible routes of contamination by MDR pathogens) must be the basis for creating a risk assessment (RA) framework [23,24]. Animal products (meat, eggs, and milk) are proven to be a key route for *E. coli* and *Salmonella* (extraintestinal MDR pathogens), which poses a very high risk to consumers [25,26,27]. Every year, human infections with MDR bacteria lead to a fatal outcome (death) in 700,000 people, over 90% of whom are in low- and middle-income countries. Assumptions and calculations indicate that by 2050, AMR is expected to be responsible for 10 million deaths each year, with economic consequences similar to those caused by the global financial crisis of 2008–2009 [28].

## 2. Bacterial AMR in Food-Producing Animals (FPA)

Lechner et al. [29] reported that resistant bacteria can be transmitted to the human population from animal sources through direct contact, indirectly through food, or via water (from the environment). These also include poultry, pigs, and cattle as the most dominant meat sources. Antibiotic resistance develops because antibiotics predominantly kill fewer resistant bacteria, while more resistant bacteria reproduce without competition and transfer their genes that confer resistance [30].

Growth-promoting antibiotics (GPAs) are most often administered at subtherapeutic doses, making them particularly suitable for developing resistance in bacteria [31]. In EU countries, the use of GPAs has been banned since 2006, meaning antibiotics can only be used for the prophylaxis and treatment of animal diseases [32]. Despite the ban, the use of antibiotics remains significant even in EU countries. Tang et al. [33] published a systematic review and meta-analysis summarizing the effects of interventions to reduce antibiotic use in FPA on the presence of antibiotic-resistant bacteria in animals and humans. Coordinated efforts across multiple sectors are the only path to a sustainable solution to the emergence of AMR, a critical global public health challenge [34].

In a multidisciplinary study, Ferri et al. [35] determined the diffusion of resistant bacteria and selected antibiotic resistance genes (ARGs) in antibiotic-free and conventional broiler farms in Italy. Among the Gram-negative isolates, *Aeromonas*, *Salmonella*, *Proteus* spp., and *Escherichia coli* were confirmed, and among the Gram-positive bacteria, *Enterococcus* strains were confirmed. Phenotypic and genetic analysis revealed patterns of multiple AMR to antibiotics in *Salmonella*, *E. coli*, and *Serratia* isolates, and then in *Enterococcus* species. The antibiotic-free and conventional systems did not differ significantly in the distribution of resistance genes at the farm level, nor in the phenotypic resistance profiles of bacterial strains from both groups of samples. Thus, a weak influence of the farming model on the spread of AMR to antibiotics in the poultry meat production chain was established.

A recent review by Musuka et al. [36] reveals that in the South African beef value chain, the overuse and misuse of AM agents, imperfect production practices, informal market structures, and poor environmental management significantly contribute to the growing threat of AMR. This has facilitated the emergence and spread of resistant pathogens that threaten food safety and public health. It highlights the urgent need for an integrated “One Health” approach that has the potential to improve regulatory oversight and management of AM agents. Improving infrastructure and capacity, as well as formalizing markets, require urgent solutions without which regional sustainability of the beef industry and food security cannot be achieved.

AMR bovine pathogens can also complicate the prevention and treatment of infectious diseases in beef cattle, threatening the efficiency and profitability of beef cattle production systems. The transfer of AMR genes to pathogens that cause human diseases associated with cattle has been identified as a serious public health problem [37].

The mentioned definitions of MDR can be applied to AM agents used to treat bovine respiratory disease (BRD) caused by *Mannheimia haemolytica*, *Pasteurella multocida*, and *Histophilus somni*. The same criteria can also be applied to pigs (swine respiratory disease—SRD, caused by *Actinobacillus pleuropneumoniae*, *P. multocida*, and *Streptococcus suis*), as well as to AM agents used to treat canine skin and soft tissue infections (SSTIs) caused by *Staphylococcus* and *Streptococcus* species.

Poultry production systems strongly drive the dynamics of AMR, ultimately requiring targeted interventions to prevent this public health nightmare. Szoke et al. [34] found that Hungary uses veterinary antibiotics moderately (69.9 mg/PCU) and can serve as a valuable case study in the European context, in line with the European Food Safety Authority (EFSA) guidelines. The study focused on broilers and ducks, examining shortcomings in AMR studies (e.g., resistance analyses and detailed comparison of resistance distribution between the two production systems) while considering key differences that are influenced by farming practices. Prophylactic action through judicious use of antibiotics significantly reduces the abundance of MDR bacteria, favoring rational, evidence-based antibiotic management practices. The breeding phase is crucial for mitigating AMR. Environmental biomarkers are valuable tools for monitoring the spread of AMR.

## 3. Foodborne Infections and AMR in Meat and Meat Products

The routes of transmission of MDR pathogens from primary production to consumers (through food, along the food chain) are not fully understood, though some studies describe potential routes. MDR pathogens from primary production can reach and persist in food business operators and contaminate or recontaminate, i.e., survive and/or grow, in food or food environments, e.g., in raw foods or in ready-to-eat products at the consumption stage [38]. Minimal food processing under sublethal conditions may predispose to the horizontal transfer of plasmids with AMR genes. Heat treatment at sublethal temperatures may reduce phenotypic resistance, or it may be increased by increasing the salt concentration or decreasing pH [39].

Buncic et al. [40] published significant results of an EU research project (“ProSafeBeef”), primarily a longitudinal integrated approach (from farm to fork), to investigate the occurrence of foodborne pathogens in the beef production chain. There is no simple winning combination that would reliably and completely eliminate pathogens from the chain (“one intervention—one point in the chain”), i.e., complete prevention of the occurrence of pathogens in beef and its products at the time of consumption. Therefore, a series of control interventions at multiple points in the chain are required, which would lead to an acceptable, final reduction of the risk. Proven and recommended new interventions suggest the implementation of measures on farms, risk categorization of livestock for slaughter, a higher level of hygiene, the application of antimicrobial treatments on skin and/or carcasses during slaughter and during the processing–storage-distribution of beef, the use of safety indicators based on the integrator of time and temperature, and effective sanitation of surfaces.

AMR associated with food-borne zoonotic pathogens (infectious agents that can be transmitted from animals to humans through the consumption of food) has been recognized as a major public health concern in Europe over the last decade. Modern animal feed incorporates large amounts of antibiotics for (1) therapeutic purposes, (2) preventive purposes, and (3) growth promotion, which allows for the development of bacterial resistance to AM drugs [6,41]. Cattle, sheep, pigs, and poultry, as food-producing animals, are important for the emergence and transmission of AMR that can be expected to be transmitted to humans [8]. If antibiotics are used in one sector or compartment of the environment or country, they can certainly influence the spread of resistance in others. Commensal bacteria (*E. coli*, enterococci) also acquire AMR in response to selective pressures and become a reservoir of resistance genes in the environment, with the potential to transfer resistance to pathogenic bacteria that can lead to difficult or incurable human diseases [8].

The European Medicines Agency (EMA) defines a Maximum Residue Limit (MRL) as an acceptable concentration of residues in food products, and the European Union requires that foods do not contain residues of veterinary medicines above the MRL. The European Union (EU) legally requires that foods like meat, milk, or eggs not contain residue levels of veterinary medicines or biocidal products that could endanger the consumer’s health. Regulation (EC) No 470/2009 of the European Parliament and of the Council (EMA Regulation (EC) No. 470/2009) [42] defines rules for setting maximum permissible levels (MRLs), measured in milligrams per kilogram for solid products and milligrams per liter for liquids [43]. Antibiotics can accumulate in tissues such as muscles and organs, and their residues act as selection factors that promote the development of resistance in the microorganisms present [44].

Drastic methods with strong lethal treatments and bacterial death do not ensure that the transmission of AMR is completely excluded, as DNA from lysed cells can be transferred to living microbes on food or in the human digestive system by transformation. Environmental stress can encourage pathogen adaptation through resistance gene expression, higher virulence, and infectivity [45].

In Table 1, Wiśniewski et al. [46] presented the influence of food-processing technology on the antibiotic content in meat products. It can be concluded that the effectiveness of reducing the residual amount of antibiotics in meat depends on the processing method, the duration of the process, and the type of antibiotic. Antibiotic residues in meat lead to serious drawbacks in the production of fermented meat products, because industrial starter cultures for fermented meat products can be sensitive to antibiotic residues and alter the fermentation process, which not only changes the sensory properties of the product but also poses a risk to public health.

Dixit et al. [47] cultured bacteria on selective media from raw chicken (*n* = 244) and pork (*n* = 160) meat samples obtained from all four major supermarket chains in Australia, with the aim of determining the diversity of MDR bacteria present in meat in retail stores in Australia. Antimicrobial susceptibility testing (AST) was performed for thirteen critically important and four very important antibiotics detected in the meat samples, according to the World Health Organization (WHO) categorization. The presence of AM drug resistance genes, virulence genes, and plasmids was identified in a total of 288 isolates by whole genome sequencing (WGS). A total of 12% of the isolates (35/288) were proven to be MDR by AST. Using WGS data, 81% of isolates (232/288) were found to contain resistance genes to critically or very important antibiotics. Thus, this study reveals a greater diversity of AMR genes in bacteria isolated from retail meat in Australia than previous studies have shown, emphasizing the importance of monitoring AMR in both foodborne pathogens and other species that can transfer AMR genes to pathogenic bacteria.

Gousia et al. [48] studied the contamination of bacteria-resistant pathogens in northwestern Greece over a 3-year period, in raw meat (sheep (40), goat (40), pork (120), beef (80), and chicken (19)) and processed meat products (turkey filets (33), salami (8), ready-made minced meat (16), stuffing (22), and roast beef (50)) from retail outlets. The susceptibility of the isolated pathogens to 19 AM agents used in humans was assessed. Out of 428 samples, 157 isolated strains belonged to *Escherichia coli*, 25 *Yersinia enterocolitica*, 57 *Staphylococcus aureus*, 57 *Enterococcus* spp., 4 *Salmonella* spp., and 3 Campylobacter jejuni. Among the isolates, 14.6% of *E. coli*, 10.5% of *S. aureus*, 4% of *Y. enterocolitica*, 25% of *Salmonella* spp., and 42.1% of *Enterococcus* spp. were susceptible to antibiotics. *E. coli* from chicken showed high rates of resistance to ciprofloxacin (62.5%), followed by lamb/goat (10.9%), pork (15.7%), and beef (27.9%). Resistance to nitrofurantoin was dominant among isolates from lamb/goat (60%). Dominance of tetracycline resistance was detected in pork (68.2%) and chicken (62.5%), while resistance to aminoglycosides dominated among isolates from lamb/goat. *S. aureus* resistance to clindamycin was predominant in isolates from lamb/goat meat (50%). Resistance to ciprofloxacin was predominant in strains from swine, with no resistance to methicillin. *Y. enterocolitica* showed high ampicillin resistance (96%), and all *C. jejuni* isolates were resistant to ampicillin, cephalothin, and cefuroxime. Meat may be a source of resistant bacteria, which could potentially spread through the food chain in the community.

During the slaughter process, meat products are cross-contaminated with surrounding contact surfaces, so Tagar and Qambrani [49] determined the level of microbial contamination of poultry meat and contact surfaces in 38 poultry slaughterhouses in the districts of Hyderabad and Jamshoro, Pakistan. Samples were collected from each slaughterhouse for testing using culture-based techniques for the presence of three bacteria: *Salmonella*, *Shigella*, and *Escherichia coli*. Antibiotic resistance was also determined for *Salmonella*. *Shigella* was present in 86.8% of knife samples, 94.7% of cutting boards, and 97.3% of meat samples. *E. coli* was detected in 26 samples, 86.8% of knife samples, 97.3% of cutting boards, and 97.3% of meat samples. *Salmonella* was detected on knives in 97.3% of samples, on 92% of cutting boards, and in 97.3% of meat samples. All *Salmonella* isolates were resistant to one or more antibiotics. Resistance to ampicillin, gentamicin, cefotaxime, erythromycin, neomycin, streptomycin, and sulfamethoxazole was found in 91.7%, 25.6%, 32.1%, 40.3%, 33.9%, 34.8%, and 52.2%, respectively. Only 6.4% of isolates were resistant to azithromycin (6.4%), and none were resistant to ceftazidime. Multidrug resistance (MDR) was demonstrated in 61.4% (67/109) of isolates. The high prevalence of *Salmonella*, *Shigella*, and *Escherichia coli* in meat and on contact surfaces is considered to be a major challenge and threat to human health. Creating new, improving, and developing existing strategies for preventing the risk of foodborne diseases in slaughterhouses will be of vital importance in the future.

The study by Zelalem et al. [50] aimed to assess the prevalence and AMR profile of some bacterial pathogens isolated from meat and meat products in Ethiopia. Major electronic databases and indexing services (PubMed/MEDLINE, Google Scholar, Science Direct, and WorldCat) were used to search for data (published and unpublished studies on the prevalence and AMR profiles of bacterial pathogens in meat and meat products in Ethiopia). The pooled estimate of the outcome measures was obtained using the DerSimonian-Laird random effects model (95% confidence level). The study protocol was registered in PROSPERO with reference ID: CRD42018106361. Data were retrieved from a total of 27 original studies with 7828 meat samples and included in the systematic review and meta-analysis. The cumulative prevalence of *Salmonella* spp., *E. coli* O157:H7, *Staphylococcus* spp., and *L. monocytogenes* was 9, 5, 21, and 4%, respectively. The prevalence of *Salmonella* in goat, sheep, beef, pork, chicken, and fish was 18, 6, 10, 11, 14, and 2%, respectively. The data analysis revealed a prevalence of *E. coli* O157:H7 in beef, sheep, goat, and other animal meats of 6, 6, 3, and 21%, respectively. The prevalence of *Staphylococcus* spp. in beef and other animal meat was 21% and 22%, respectively. Based on the origin of the sample, the prevalence of *Salmonella* in the slaughterhouse, butcher’s shop, and market was 6, 36, and 11%, respectively. For *E. coli* O157:H7, the prevalence in the slaughterhouse, butcher’s shop, and market was 5, 6, and 8%, respectively. The bacterial isolates showed different AMR profiles to the selected drugs (about 25% of *Salmonella* spp. showed resistance to ampicillin, 9% of *Salmonella* spp., and 2% of *E. coli* O157:H7 were resistant to ceftriaxone). The pooled estimates revealed that 10% of the *E. coli* O157:H7 isolates were resistant to ciprofloxacin. *Salmonella* spp. (6%), *L. monocytogenes* (5%), and *E. coli* O157:H7 (2%) showed resistance to gentamicin. The conclusion indicates that the cumulative prevalence of bacterial pathogens is relatively high compared to other countries, and it is recommended to design an intervention to ensure meat safety in the sector.

The potential risks of AMR in fermented foods and beverages have been the focus of a number of studies on antibiotic resistance genes in lactic acid bacteria (LAB) and coagulase-negative staphylococci, most commonly to tetracyclines, penicillins, chloramphenicol, and macrolides [51,52,53]. The review paper by Fraqueza [54] is very useful for learning about the issue of antibiotic resistance in LABs isolated from fermented meat products (sausages). The conclusion of the original scientific paper by Leroy et al. [55] is that there is a possibility of transmission of tetracycline resistance between *S. xylosus* strains in fermented sausages, with a very low frequency demonstrated in vitro and in situ. Based on the findings of Qu et al. [56], in addition to the pathogens most commonly transmitted by food, the assessment of the microbiological safety of fermented meat products must include the detection and control of coagulase-negative staphylococci, *Acinetobacter*, and *Pseudomonas* in order to address the problem of bacterial resistance. A convenient and rapid method for assessing the total amount of basic resistome is the quantitative detection of *lnuA* and *abeM*, which is important for the control of bacterial resistance and the prevention of the occurrence of pathogens in fermented meat products.

Shahraz et al. [57] examined a total of 256 packaged hamburgers in Tehran, Iran, for the presence of *Staphylococcus aureus*, using the standard disk diffusion method to detect the antibiotic resistance pattern, and detected the *mecA* gene in the isolated strains using a sensitive and specific molecular technique, PCR. *S. aureus* was positive in 25% of the samples. Resistance to methicillin, erythromycin, penicillin G, cefazolin, ciprofloxacin, vancomycin, and amoxiclav was found in 89%, 20.3%, 18.7%, 15.6%, 14%, 26.6%, and 12.5%, respectively. The *mecA* gene was present in 100% of resistant isolates, 0% of intermediate-resistant isolates, and 25% of susceptible isolates, as confirmed by the PCR analyses of methicillin-resistant *S. aureus* (MRSA). The results of the PCR-based detection of the *mecA* gene confirmed a high correlation with the results of the standard disk diffusion test.

### 3.1. AMR Pathogens Transmitted by Meat and Meat Products

In this section, we explore how to make a gradation of bacterial agents, the causes of foodborne diseases, according to their danger to human health, their frequency of occurrence, and the level of their resistance.

#### 3.1.1. *Salmonella*

We will start with the fact that one of the most common causes of foodborne bacterial diseases in humans is certainly *Salmonella* [58]. The poultry industry and regulatory authorities are under intense pressure due to the looming threat of the emergence of *Salmonella* contamination, which they consider the leading global cause of foodborne infections [59,60]. The public health challenge is even greater when antibiotic-resistant *Salmonella* isolates are discovered [61]. Poultry is the primary reservoir of *Salmonella*, a potential contaminant of chicken meat and eggs [62]. The great challenge of pathogen carriage and the difficulty in eradicating *Salmonella* from reservoirs requires an attack on *Salmonella* spp. in the animal feed supply chain, with the implementation of rigorous preventive measures as a precaution and the development of improved modern therapeutic strategies in order to minimize the hazard to public health [63]. Foodborne transmission of *Salmonella* spp. is most common with chicken meat [64]. In 25% of cases, salmonellosis is transmitted through contaminated raw poultry [62,65], while other routes of transmission include unhygienic farming, contaminated food, slaughter, processing, and packaging practices. Predisposing factors that increase the risk of infection are inadequate or prolong transportation, storage, retailing, and food handling or preparation by consumers.

A number of recent studies have shown that the emergence of resistance to the most commonly used antibiotics against *Salmonella* spp. isolated from animals or humans is progressively increasing [58,66,67]. Cunha-Neto et al. [68] reported that multidrug-resistant (MDR) isolates of *Salmonella* spp. that cause serious infections contain several genes encoding antibiotic resistance: *bla*_TEM_ (beta-lactam resistance gene), *sul*1 and *sul*2 (sulfonamide resistance genes), and *tet*B (tetracycline resistance gene). A global challenge for human public health is the emergence of new antibiotic resistance in foodborne pathogens, e.g., in *Salmonella*, as AMR bacteria cause life-threatening disease (700,000 deaths worldwide, with 23,000 deaths in the United States, annually) [69,70]. In the United States, *Salmonella* was detected in 8.6% of chicken breast samples; resistance to more than one antibiotic was detected in more than 73.1% of isolated *Salmonella* spp., while resistance to more than three types of antibiotics (MDR) was demonstrated in 48.1% of isolates [71]. Zaiko et al. [72] investigated the prevalence, serovars, and AMR profile of *Salmonella* isolated from meat and minced meat intended for the production of fermented sausages. A total of 116 samples were tested, and 20 were positive (17.2%). *Salmonella* was detected in three (10.3%) beef samples, five (19.2%) pork samples, and six (20.7%) poultry samples. The prevalence of *Salmonella* in minced meat was 18.8%. The most frequently isolated serovar was *Salmonella enterica* serovar Agama (5.2%), followed by *S. Enteritidis* (4.3%), *S. Typhimurium* (3.4%), *S. Infantis* (2.6%), and *S. Lindenburg* (1.7%). Most of the listed serovars are recognized as common causes of salmonellosis in humans, which means that food containing this meat is a potential source of human infections. *Salmonella* isolates showed high rates of resistance to tetracycline, ampicillin, streptomycin, and ciprofloxacin, with the highest level being to tetracycline (75%), followed by resistance to ampicillin (50%), streptomycin (30%), ciprofloxacin (20%), gentamicin (20%), and neomycin (10%). MDR *Salmonella* in meat used for the production of fermented sausages additionally poses a high risk to human health.

In the most recent research, Al-Qadiri et al. [63] determined and published different patterns of prevalence of *Salmonella* spp. detected in raw chicken meat. There is a very high risk of contamination of chicken with *Salmonella* due to the fact that it can come from numerous sources of contamination during poultry farming, slaughtering, processing, and marketing activities. The emergence of AM-resistant strains ultimately requires that a strategy to achieve *Salmonella*-free raw chicken meat be introduced as a priority. *Salmonella* isolates showed phenotypic resistance to beta-lactams, sulfonamide, tetracycline, and ciprofloxacin. Antimicrobial resistance genes (RGs) *bla*_TEM_, *tet*(B), *sul*1, and *sul*2 were detected among *Salmonella* isolates. Genotypic results follow phenotypic AST. Therefore, raw broiler chicken is a major source of MDR *Salmonella*. It can be concluded that strict control of antibiotic use is reasonable, because uncontrolled use or misuse of AM agents in livestock farming can cause the re-emergence of MDR bacteria that could otherwise lead to higher rates of morbidity and mortality due to foodborne diseases (FBDs) caused by *Salmonella*.

The increasing incidence of gastroenteritis (GE) and sepsis caused by resistant–MDR strains of non-typhoidal *Salmonella* (NTS) and to commonly used antibiotics (beta-lactams, aminoglycosides, and quinolones) is attracting the attention of health authorities and decision-makers. The production of β-lactamase by *Salmonella* results in resistance to ampicillin and cephalosporins, and mutations in certain genes (*gyr*A and *par*C) result in resistance to ciprofloxacin. Low levels of resistance to quinolones and efflux pumps are mediated by plasmids. AMR has been observed to vary in different regions, with MDR in Europe being detected at 22.6%, ampicillin at 25.2%, third-generation cephalosporin resistance at 1.1%, and fluoroquinolone resistance at 14.9%. In China, resistance rates to MDR were found to be 40–81%, 73.4% to ampicillin, 20% to third-generation cephalosporins, and 16.2% to fluoroquinolones. The United States has MDR rates of 10.3%, ampicillin resistance rates of 6.6%, third-generation cephalosporins resistance rates of 3%, and fluoroquinolones resistance rates of 3% [73].

During official control and internal control procedures in 2015 in Latvia, Terentjeva et al. [74] tested a total of 3152 samples of raw and ready-to-eat (RTE) meat for the presence of *Salmonella*, in accordance with ISO 6579:2002 [75]. The prevalence of *Salmonella* was 0.8% (25/3152), with the highest prevalence (1.5%) in minced meat and meat products (7/481) and the lowest (0%) in frozen meat and meat products (0/349) and RTE meat (0/364). *S. Typhimurium* (36%, 9/25) and *S. Derby* (32%, 8/25) were most frequently detected. The percentage of *Salmonella* isolates resistant to at least one antimicrobial agent was 62% (13/21), of which 40% (8/20) were resistant to sulfamethoxazole; 25% (5/20) to nalidixic acid, ciprofloxacin, and ampicillin; and 20% (4/20) to tetracycline. All isolates were susceptible to ceftazidime, cefotaxime, meropenem, azithromycin, and tigecycline. *S. Typhimurium* showed AMR more often (87.5%) than other strains. All results indicate the high importance of the problem of the presence of *Salmonella* in meat, as the high prevalence of resistant strains poses a public health threat in Latvia.

Kalchajanand et al. [76] tested 68 non-AMR *Salmonella* strains treated with half the concentrations of lactic acid (LA), peracetic acid (PAA), and cetylpyridinium chloride (CPC), which are applied in beef-processing plants to monitor tolerant strains. A challenge study was conducted with six *Salmonella* strains, each of which, from the non-AMR *Salmonella* strains that were most tolerant to LA (2%), PAA (200 ppm), and CPC (0.4%), were selected and inoculated onto fresh beef surfaces and subjected to spray-washing treatments (4% LA, 400 ppm PAA, or 0.8% CPC). Tissue samples were collected before and after each AM treatment to count surviving bacterial colonies. Spray treatment with LA, PAA, or CPC significantly reduced non-AMR *Salmonella* and AMR *Salmonella* on treated beef surfaces by 1.95, 1.22, and 1.33 log CFU/cm^2^; and 2.14, 1.45, and 1.43 log CFU/cm^2^, respectively. The efficacy was in the ascending order: LA ≤ PAA = CPC. LA, PAA, and CPC were equally effective against non-AMR and AMR *Salmonella* on fresh beef surfaces (*p* < 0.05). The significance of the results of this study is the conclusion drawn from them that foodborne pathogens that have acquired both antibiotic resistance and antimicrobial tolerance are still equally susceptible to AM treatments during meat processing.

#### 3.1.2. *Campylobacter* spp.

Jung et al. [77] in his study in Table 1 presented a summary of global AMR surveillance programs targeting food, animal, and retail meat bacterial isolates, so it can be seen that *Campylobacter* is the subject of surveillance in all countries of different regions (Europe, North America, Asia/Pacific, and South America) that are included in the review, as the most common zoonotic foodborne pathogen, with 58.9% of confirmed cases in humans [78] or 45.7 reported cases per 100,000 population. The zoonotic potential of drug-resistant *Campylobacter* is reflected in the fact that it can be transmitted to humans through contaminated food, water, and direct contact with animals [79]. The primary cause of Campylobacteriosis in humans is the consumption of undercooked poultry meat. Epidemics occur after the consumption of raw, uncooked milk or dairy products made from such milk. *Campylobacter* can be transmitted to humans from pets (dogs and cats), as it is present in their intestines, and interaction with puppies infected with *Campylobacter* allows its transmission. Exposure of *Campylobacter* to antibiotic treatments (in humans, FPA, and pets) favors the development of various resistance mechanisms in this bacterium. Horizontal gene transfer (HGT) plays a pivotal role in disseminating resistance genes among *Campylobacter* populations. Mechanisms such as conjugation, transformation, and transduction facilitate the acquisition of resistance determinants from other bacterial species, further accelerating the spread of AMR [80]. Antibiotic-resistant *Campylobacter* is increasingly diagnosed, reducing the effectiveness of antibiotic treatment, which is a significant risk that threatens public health. Increasing rates of AMR to macrolides (which are highly preferred in the treatment of *Campylobacteriosis*) are observed, especially in *Campylobacter coli* strains from China, Spain, and Peru, where they are the “drug of choice” for the treatment of campylobacteriosis [81]. Alternatives to macrolides are contraindicated in children (fluoroquinolones and tetracycline). Erythromycin resistance is relatively low in humans in Europe, whereas a rapid increase in AMR to ciprofloxacin and tetracycline in *Campylobacter* spp. has been detected. The prevalence of fluoroquinolone resistance is 75–90% in clinical *Campylobacter* strains in different countries [82,83]. Aminoglycosides (gentamicin) are effective in treating systemic infections. Due to the increasing incidence of MDR *Campylobacter* spp., there are initiatives to include amoxicillin–clavulanic acid or Fosfomycin tromethamine as treatment options (after studies evaluating their efficacy). Macrolides- and fluoroquinolones-resistant *Campylobacter* strains are on the rise in FPA, like ciprofloxacin-resistant *Salmonella* [84].

Biosecurity and immune-based methods (pre-harvest strategies) reduce bacterial burden on farms, and decontamination and freezing of carcasses (post-harvest measures) limit contamination. Alternative chemical-free approaches are promising (application of bacteriocins and natural antimicrobials) [85,86]. Multidisciplinary interventions integrated throughout the food chain are necessary to improve global public health protection: antimicrobial stewardship, implementation of sustainable agricultural practices, and innovative solutions [79].

#### 3.1.3. Diarrheagenic *E. coli*

In the research of Madhup et al. [87], the most abundant microorganism in meat samples was *E. coli*. Also, drug MDR was most common in *E. coli*, with resistance to ampicillin, ciprofloxacin, and gentamicin. More controlled use of antibiotics in the animal husbandry industry and a more hygienic environment in meat vendors are necessary. If *E. coli* is detected in meat samples, it can serve as an indicator of (low) food hygiene, and the presence of AMR strains of *E. coli* indicates the risk of infection in humans [88]. In the research of Madhup et al. [87] on the prevalence of pathogenic bacteria in meat products and their AMR pattern, *E. coli* was the dominant bacterium in meat samples. Resistance against ampicillin, ciprofloxacin, and gentamycin MDR was also most frequent in *E. coli*.

#### 3.1.4. Shiga-Toxin-Producing *E. coli* (STEC) Infections

Use of third- and fourth-generation cephalosporins against *E. coli* infections in livestock was linked to resistance to the same bacteria found in humans. A study conducted by Gweshe et al. [89] aimed to (A) assess *E. coli* contamination in polony, beef burgers, and traditionally fermented cow milk from the formal and informal markets in Harare, Zimbabwe; (B) determine the antibiotic sensitivity of *E. coli* isolates; and (C) identify Shiga-toxin-producing *E. coli* isolates using the presence of virulence genes (intimin, enterohemolysin A, and Shiga toxins 1 and 2). From informal and formal outlets of the central business district, 96 samples comprising 32 beef polony slices, 32 beef burger patties, and 32 fermented milk specimens were obtained. *E. coli* occurred in 20 (21%) of the samples, being more prevalent in the informal (29%) than in the formal (13%) market. Of the 20 *E. coli* isolates, 6 (30%) were Shiga-toxin-producing *E. coli*, and the rest (70%) were negative for virulence genes. The predominance of *E. coli* was greater in meat products (25%) than in fermented milk (13%). Total *E. coli* counts were not substantially different between formal and informal markets (*t*-test: *p* = 0.08). All the *E. coli* isolates were MDR, with AM resistance prevalence ranging from 25% for Sulphamethoxazole to 100% for Penicillin and Erythromycin. The presence of *E. coli* in food indicates fecal contamination and probable existence of other enteric pathogens. The presence of virulent and AMR *E. coli* strains in food threatens food safety and public health. Ready-to-eat animal products from both sectors (informal and formal) could result in the dissemination of AMR *E. coli* species if corrective measures are not predicted and taken.

#### 3.1.5. *Listeria monocytogenes*

The acceleration of AMR of *Listeria monocytogenes* (*L. monocytogenes*) is associated with increased global trade and transport, which facilitates the spread of AMR between countries and continents. The excessive prescription and use of drugs (antibiotics) in therapy or their increasingly limited use as growth promoters in FPA leads to AMR [90,91]. *L. monocytogenes* strains resistant to first-line antibiotics have been isolated and described, as well as clinical strains resistant to gentamicin [92], and an ampicillin-resistant strain has been identified in the USA [8]. Clinical isolates of *L. monocytogenes* from different countries are resistant to streptomycin, erythromycin, kanamycin, sulfonamide, and rifampin [89,90]. MDR to antibiotics has been identified in environmental samples as well as in strains isolated from food worldwide [93]. In Northern Ireland, Walsh et al. [92] found that 0.6% of *L. monocytogenes* were from retail foods. AMR for *L. monocytogenes* isolated from food and animal sources (*n* = 167) in the USA was determined to be 1.8%, 9%, 73%, and 100% to ciprofloxacin, tetracycline, sulfonamide, and nalidixic acid, respectively [90]. *L. monocytogenes* becomes resistant to AMA agents by acquiring three types of mobile genetic elements (self-transferring plasmids, mobilizing plasmids, and conjugative transposons) [91]. Efflux pumps are thought to be the mechanism of resistance to fluoroquinolones in *Listeria* [94]. There is increasing evidence of spontaneous acquisition of resistance genes by *L. monocytogenes* [91], in promoter- or operator-coding regions, which can overexpress endogenous genes, such as those encoding AM inactivating enzymes, such as the β-lactamase AmpC gene [95]. Point mutations occurring in genes encoding AM target regions can result in a target site that is resistant to AM activity, such as in the gene gyrase, whose mutation leads to the expression of a fluoroquinolone-resistant gyrase enzyme [96].

#### 3.1.6. *Staphylococcus aureus* (SA)

SA is a common pathogen in both humans and FPA, with high levels of antibiotic resistance. Globally, the number of deaths associated with or attributed to antimicrobial resistance (AMR) caused by methicillin-resistant SA has increased the most, from 261,000 associated deaths and 57,200 AMR-attributable deaths in 1990, to 550,000 associated deaths and 130,000 AMR-attributable deaths in 2021 [97]. Haskell et al. [98] examined the prevalence of SA and MRSA in conventional and antibiotic-free (AF) meat products and determined antibiotic resistance rates in isolates obtained from raw conventional turkey, chicken, beef, and pork, as well as from AF chicken and turkey samples. Contamination in conventional meat with SA was common (prevalence of 22.6%, range 2.8–30.8%) and stood at 13.0% (level 12.5–13.2%) in AF poultry meat. MRSA was isolated from 15.7% of conventional raw meat (2.8–20.4%) but was not isolated from AF meat. The level of antibiotic resistance in conventional poultry products was significantly higher compared to AF-resistant poultry products for a number of different antibiotics, and while multidrug-resistant strains were relatively common in conventional meat, none were detected in AF-resistant meat.

Currently, there are AM agents available for the treatment of bovine respiratory disease (BRD) caused by *Mannheimia haemolytica*, *Pasteurella multocida* and *Histophilus somni*, classified into seven classes of drugs (which include the aminocyclitol, penicillin, cephalosporin, fluoroquinolone, macrolide, phenicol, and tetracycline classes). There are also seven classes of drugs available for the treatment of swine respiratory disease (SRD) caused by *Actinobacillus pleuropneumoniae* (penicillin, cephalosporin, fluoroquinolone, macrolide, phenicol, pleuromutilin, and tetracycline classes), six for *Pasteurella multocida* (penicillin, cephalosporin, fluoroquinolone, macrolide, phenicol, and tetracycline classes), and five for *Streptococcus suis* (penicillin, cephalosporin, fluoroquinolone, phenicol, and tetracycline classes). Specific clinical breakpoints have been determined for the categorization of isolates as MDR, XDR, or PDR.

There are agents available for the treatment of skin and soft tissue infections (SSTI) in dogs in six classes of drugs caused by *Staphylococcus* spp. (aminoglycoside, penicillin, cephalosporin, fluoroquinolone, tetracycline, and lincosamide classes) and five classes available for *Streptococcus* spp. (classes of aminoglycosides, penicillins, cephalosporins, fluoroquinolones, and lincosamides). Therefore, MDR, XDR, and PDR can also be defined for pathogens not listed above.

## 4. Transmission of AMR to the Human Population

The development of AMR in the human population is still enigmatic. There are no completely precise data on the impact of antibiotic prevention (e.g., metaphylaxis) or random or targeted therapy (according to in vitro various AM susceptibility test results) in livestock or in the primary Agri-Food sector and the importance of the food chain in the selection and transmission of resistant genes and/or bacteria [7,23,99]. The transmission of MDR foodborne pathogens (through the food chain) from animals to humans is confirmed by the fact that most *Salmonella* isolates are MDR (*S. enterica* serovar Muenchen) because they carry a newly discovered megaplasmid (*S. infantis* pESI) that confers resistance to a larger number of antibiotics [100]. Antibiotic therapy becomes ineffective for treating patients due to AMR, and accordingly, the resistance of *Salmonella* isolates should be monitored [101].

Recently, nosocomial infections with high mortality rates due to the spread of ESKAPE pathogens (*Enterococcus faecium*, *Staphylococcus aureus*, *Klebsiella pneumoniae*, *Acinetobacter baumannii*, *Pseudomonas aeruginosa*, and *Enterobacter species*) have been described, against which the biocidal action of several species or classes of antibiotics will not exert an AM effect, both in humans and in animals.

Given that infections with MDR organisms can endanger the population of healthy people, examples of the serious impact of MDR on the deterioration of the health status of the population of sick people (e.g., from acute myeloid leukemia—AML) are also very significant, as can be seen in the recent study by Trajković et al. [17]. This work indicates a high incidence of infections in this population, especially with MDR and XDR strains of *Klebsiella* spp. and *Enterococcus* spp., as well as a high rate of early death. The mortality rate of patients diagnosed with MDR bacterial infection was very high (36.7%).

## 5. Strategies to Prevent the Occurrence of AMR Microorganism Infections

Effective alternatives to the preventive and therapeutic use of antibiotics must be identified, the application of which would allow for a further trend of increased presence of animal products on the market. Alternatives must allow for a reduction in the use of antibiotics in maintaining animal health without significantly increasing the rate of infectious diseases that harm them. The authors propose that such alternatives be designed and financed from the revenue from taxes on meat produced using antibiotics [32]. In his study, Fumagalli [102] states that state intervention that restricts consumer freedom would be legitimate because individual cases of meat consumption or purchase can significantly contribute to the development of AMR in agricultural environments. Integrated agricultural groups are required to reduce the use of AM agents through the targeted application of safe vaccines, plant-based veterinary drugs, enzymes, and other agents [103]. Intervention strategies to address the global challenge associated with AMR are presented in Table 1. Vaccination to prevent infections that would otherwise have to be prevented or treated with antibiotics is the first alternative strategy. WHO defines that “sustainable animal husbandry practices, including the use of vaccines, can reduce infection rates and antibiotic dependence, as well as the risk that antibiotic-resistant organisms will develop and spread through the food chain” [104]. Another alternative strategy may be to reduce antibiotic use through the implementation of stocking density standards or regulations in livestock facilities.

Using nutritional (natural) supplements instead of antibiotics is the third strategy. The superiority of biological control methods is reflected in the wide spectrum of antimicrobial (AM) activity, circumvention, and avoidance of the development of microbial resistance. Natural AM agents are biodegradable, with low allergenic potential. The most commonly tested and used means for biological control are natural extracts, primarily of plant origin but also of animal or microbial origin [105]. In food processing, with the modern trend to produce healthier, “green”, or functional food, which could be declared as a “clean label”, the use of biological control methods has a slight but continuous upward trend because they have natural and environmentally friendly properties and, above all, because they are safe [106].

A fourth alternative strategy is that of breeding animals with better immunity (better adapted breeds) to common diseases, which would reduce the need for therapeutic use of antibiotics (lower level of mortality, improved animal health and welfare, and lower costs of expensive antibiotics and veterinary services).

Resistant bacteria can infect humans either by transmission through contact with animals or consumption of animal products [30,107]. Livestock farmers who raise animals infected with methicillin-resistant SA are at higher risk of infection with these bacteria than if they were not exposed to animals as a source of resistant bacteria [104]. The WHO defines in its global action plan on AMR that “food is one possible means of transmitting resistant bacteria from animals to humans, and human consumption of food containing antibiotic-resistant bacteria has led to the acquisition of antibiotic-resistant infections” [104].

In order to protect the efficacy of important AMDs for human use, the WHO has published guidelines providing evidence-based recommendations and best practice statements on the use of “medically important antimicrobials” in animal feed, defined as AM classes used in human medicine. Medically important AMDs are categorized as “important”, “highly important”, or “critically important” for human medicine according to appropriate criteria. The WHO recommends that the overall use of medically important AMDs in animal feed be reduced. Their use for growth promotion in animals without a diagnosed clinical disease is prohibited. The WHO further recommends that critically important AMDs be used only for the treatment of specific animal diseases, and that critically important AMDs, the highest priority, are prohibited for use as additives in animal feed (WHO, 2017) [108].

Giubilini et al. [32] propose in their scientific paper, as a feasible and fair way to address the problem of antibiotic resistance, the taxation of animal products obtained through the use of antibiotics, as such (often uncontrolled) use in livestock farming is one of the main causes of antimicrobial resistance (AMR) in both animals and humans. There are no precise data on the global consumption of antibiotics in livestock farming, but estimates range from 63,000 t to 240,000 t [109]. The large-scale use of antibiotics in animals is not therapeutic; antibiotics are often given for prophylactic (metaphylaxis) or production purposes (i.e., growth promoters) [110]. Estimates vary considerably, especially in developing countries, due to inadequate surveillance and data collection [111]. European authorities have published an estimate that 11,381.8 tonnes of active substances with AM activity were used in humans and animals for food in 26 EU/EEA countries in 2012 [112]. Biocides are increasingly used as antibacterial agents because they are highly effective, low-toxic, and have long-lasting properties [106].

In addition to biocides, non-thermal sterilization technology ensures food safety, extends shelf life, and improves quality. It is imperative to meet consumer demand for natural and healthy food and increase its competitiveness in the market, promoting the sustainable development of the food industry. The work of Zhang et al. [106] is a comprehensive review of specific applications of biocides and non-thermal sterilization methods in food. The importance of control parameters and their impact on microorganisms during low-temperature meat processing is discussed.

Anachinaba et al. [113] conducted a useful consumer study in the Tema Metropolitan Area, Ghana. They used a semi-structured questionnaire to assess consumer knowledge and perceptions of microbiological safety of meat, antibiotic resistance, and residues. The questionnaires were presented to 384 randomly selected consumers. Data analysis was performed using the Statistical Package for Social Sciences version 20, and the Chi-square test was used to determine correlations between some parameters. The survey results revealed that 56% of the respondents were male, and 54% were aged between 21 and 40 years. A total of 51% of the respondents only had a primary school education. The majority of respondents preferred chicken (53%) over beef (32%) and pork (14%), mainly due to taste (50%), affordability (39%), and price (11%). When asked whether meat consumption is associated with hypertension/high cholesterol and diabetes, the majority of respondents (80%) agreed (ranging from mild to strong agreement). The majority of respondents (64%) had heard about the microbiological safety of meat, mainly from their school teachers (62%) and through various media (25%). The respondents were aware of antibiotic resistance (55%) and antibiotic residues (53%), predominantly from their school teachers (56% and 58%, respectively), but without in-depth knowledge of antibiotic resistance and antibiotic residues. The results of the consumer survey justify the need for their education on food safety and antibiotic-related issues.

Hinchliffe et al. [114] in their research address the role of market actors in the preventive regulation of agricultural practices due to the increasingly serious threats of the emergence of AMR. They report that the UK poultry sector has achieved impressive reductions in the use of AM agents in recent years. Interviews with farmers and veterinarians mapped the different practices used. The livestock sector has made significant progress in a short time in managing the threat of AMR associated with livestock.

## 6. AMR Surveillance in Meat Production and Processing

Jung et al. [76] tabulated a summary of global AM resistance surveillance programs targeting food, animal, and retail meat bacterial isolates in their review paper. Due to all the specificities and requirements, it is difficult for the agricultural industry to develop a feasible program to subsidize antibiotic-free treatment [115]. Yang et al. [116] published the results of their research as part of the Ecology from Farm to Fork Of microbial drug Resistance and Transmission (EFFORT) project, co-funded by the European Commission, 7th Framework Program for Research and Innovation (FP7-KBBE-2013-7, grant agreement: 613754). It is important to compare the abundance of AMR genes (ARGs) along animal production chains, which can be performed by quantitative real-time PCR (qPCR). A decrease in the number of ARGs was observed “from farm to fork”, i.e., along the pig and broiler production chains. ARGs were more abundant in farmers than in slaughterhouse workers, and the lowest number was recorded in control subjects. ARG abundance was highly correlated (Spearman’s ρ.0.7) between the data resulting from qPCR and metagenomic data of the pooled samples.

The strategically important study on AMR was published by numerous collaborators from a large number of countries and different regions around the world, who created and published it in 2024 in the influential journal “Lancet”. The study reveals the global burden of bacterial AMR from 1990 to 2021, including a systematic analysis with projections to 2050. The enormous significance of the challenge associated with AMR is evidenced by the following finding: in 2021, an estimated 4.71 million deaths (95% UI 4.23–5.19) were estimated to bacterial AMR, including 1.14 million (1.00–1.28) deaths attributable to bacterial AMR. Mortality from AMR has varied significantly over the past 31 years, most notably by age and region. Over the past two decades (1990–2021), deaths from AMR infections have decreased by >50% in children under 5 years of age, but have increased by >80% in adults aged 70 years and older. In super-regions, mortality has decreased in children under 5 years of age and increased in people over 5 years of age [97].

The designation of European Union Reference Laboratories for Public Health (EURL), one of which addresses AMR-related issues (EURL-PH-AMR), was carried out by the European Commission (EC) through Regulation 2022/2371 on “Serious cross-border threats to health”. The EURL-PH-AMR aims to provide services and support to the European Centre for Disease Prevention and Control (ECDC), the European AMR Surveillance Network (EARS-Net), and the European AMR Gene Surveillance Network (EURGen-Net).

Table 2 shows recently AMR monitoring and surveillance programs and the organizations that implement them, countries or regions where the programs are implemented, monitoring of samples originating from humans, food, or animals, as well as literature data sources (references). The need for multidisciplinary approaches to address the challenges of AMR and MDR microorganisms is also highlighted in a study by Hughes et al. [117], which examines the ecological complexity of the problem, as well as the responsibility and impact of the supply chain through a distributed group and stakeholder action in the UK. It defines how to achieve antibiotic reduction targets in domestic meat production through a specific and narrow strategic focus, while considering the geographical and biological limitations of the targets in addressing AMR as a serious problem.

In a significant review, McNulty et al. [118] presented a simple summary of selected studies related to AMR in the meat chain in a tabular format. The review describes the anti-AMR monitoring and surveillance programs in five selected EU countries and the European Economic Area (EEA). Sampling schemes, susceptibility testing for AMR identification, clinical breakpoints (clinical resistance), and epidemiological breakpoints (microbiological resistance) are discussed in an insightful and comprehensive manner. The most important variations between and within food-producing animal species, between countries, are explored in order to find the most effective model for addressing and managing AMR in the food chain.

The contribution of the meat chain to the development and transmission of AMR from food-producing animals (FPA) to humans in five selected countries (four EU Member States—Denmark, France, the Netherlands, and Sweden—and one EEA country—Norway) was assessed by reviewing the available scientific and professional literature. It was concluded that healthy or diseased FPA and the resulting meat are regularly sampled on farms, in the slaughterhouse, and in retail outlets. Significant differences were observed in the sampling and susceptibility testing schemes with regard to the FPA category, sample matrix (feces, caecum, fresh meat), and module in the meat chain (farm, slaughterhouse, retail) where sampling was carried out. In all five countries, detection and susceptibility testing for *Salmonella*, *Campylobacter*, *E. coli*, and enterococci were included in the national plan, although the choice of the FRA category, matrix, and module in the meat chain was not identical. In Denmark, the Netherlands, Sweden, and Norway, routine susceptibility testing for the main foodborne zoonotic pathogens in humans (*Salmonella typhimurium* and *Campylobacter jejuni*) was performed in blood, urine, and cerebrospinal fluid samples. The disk diffusion method is widely used in France and Sweden, while the dilution method is routinely used in Denmark, the Netherlands, and Norway. Limited data were available from France (individual studies on the AMR profile of *L. monocytogenes*, *Campylobacter*, and *Salmonella*). In addition to the above pathogens, *Yersinia enterocolitica* and *Shigella* were also regularly included in the national human AMR surveillance plan in Norway. Susceptibility testing of commensals in humans has not been systematically carried out in any of the five EU countries mentioned above [118].

Integrated monitoring and surveillance of AMR in commensal and zoonotic bacteria transmitted by food, from humans, animals, and food, is an essential source of information when formulating measures to improve food safety and protect consumers from exposure to resistant bacteria from food. In order to harmonize sampling and susceptibility testing and ensure better consistency between EU Member States, the EFSA guidelines for AMR monitoring should be implemented (e.g., target number of isolates per animal population—on farm, in slaughterhouses, and in retail; susceptibility testing method; panel of antimicrobials to be included and testing scope).

Effective risk reduction strategies to address AMR in the context of the food chain (meat) should be based on the promotion of cross-sectoral cooperation at the national and international levels. Veterinary, agricultural, and pharmaceutical authorities at the national level should consider establishing a regulatory framework for the approval and control of veterinary medicines, including critically important antibiotics for veterinary medicine and human health. Integrating monitoring and surveillance across the environment-feedstock-food (meat)-human continuum is of paramount importance for successfully addressing the problem of antimicrobial resistance.

**Table 2:** Recent AMR monitoring and surveillance programs and the organizations that implement them.

Program	Country	Type of Surveillance			Reference
		Humans	Food	Animals	
Danish Integrated Antimicrobial Resistance Monitoring and Research Program (DANMAP)	Denmark	×	×	×	DANMAP [119]. https://www.danmap.org/reports/2023 (accessed on 29 June 2025)
French surveillance network for antimicrobial resistance in pathogenic bacteria of animal origin (RESAPATH)	France	×	/	×	Anses [120]. https://www.anses.fr/en/content/anses-request-based-opinions-and-reports?titre=RESAPATH (accessed on 29 June 2025)
Monitoring of Antimicrobial Resistance and Antibiotic Usage in the Netherlands (MARAN) and NethMap/MARAN report for 2024.	Netherlands	×	×	×	NethMap [121]. https://www.rivm.nl/bibliotheek/rapporten/2024-0117.pdf (accessed on 29 June 2025)
Swedish Antibiotic Sales and Resistance in Human Medicine (SWEDRES) and Swedish Veterinary Antibiotic Resistance Monitoring (SVARM)	Sweden	×	/	×	Swedres-Svarm [122]. https://www.sva.se/en/what-we-do/antibiotics/svarm-resistance-monitoring/swedres-svarm-reports/ (accessed on 29 June 2025)
A report from five countries in the EU and EEA on AMR monitoring and surveillance in the meat chain	Denmark, France, Netherlands, Sweden, Norway	/	×	/	Mc Nulty et al. [118]. https://doi.org/10.1016/j.tifs.2016.09.010 (accessed on 29 June 2025)
ECDC/EFSA/EMA—integrated analysis of the consumption of AM agents and occurrence of AMR in bacteria from humans and FPAs	Europe	×	/	×	ECDC/EFSA/EMEA [112]. https://www.ecdc.europa.eu/en/publications-data/ecdcefsaema-first-joint-report-integrated-analysis-consumption-antimicrobial (accessed on 29 June 2025)
Norwegian Surveillance System for Antimicrobial Drug Resistance (NORM/NORM-VET)	Norway	×	×	×	NORM/NORM-VET [123]. https://www.vetinst.no/en/surveillance-programmes/norm-norm-vet-report/_/attachment/inline/78155e88-2b2e-42a1-ac0c-b06071eb0479:21bb52d0f6c051c93f0d4a0605eb001e25e7fe99/NORM%20NORM-VET%202023%20(2).pdf (accessed on 29 June 2025)
The surveillance program for methicillin-resistant *Staphylococcus aureus* in pigs in Norway 2024	Norway	/	/	×	Urdahl et al. [124]. https://www.vetinst.no/en/surveillance-programmes/mrsa-in-pigs (accessed on 29 June 2025)
Global antimicrobial resistance and use surveillance system (GLASS) report (2022).	Global (127 countries, territories, and areas)	×	/	/	WHO [125]. https://www.who.int/publications/i/item/9789240062702 (accessed on 29 June 2025)
GBD 2021 AMR Collaborators 1990–2021, with forecasts to 2050	Global	×	/	/	Naghavi, M. et al. [126]. https://doi.org/10.1016/S0140-6736(24)01867-1 (accessed on 29 June 2025)
The European AMR Surveillance Network (EARS-Net)	Europe	×	/	/	European Centre for Disease Prevention and Control [127]. https://www.ecdc.europa.eu/sites/default/files/documents/antimicrobial-resistance-annual-epidemiological-report-EARS-Net-2023.pdf (accessed on 29 June 2025)
European Centre for Disease Prevention and Control (ECDC) point-prevalence surveys	Europe	×	/	/	European Centre for Disease Prevention and Control [128]. https://data.europa.eu/doi/10.2900/5735023 (accessed on 29 June 2025)
Surveillance for AMR and healthcare-associated infections in Europe—SUSPIRE protocol: registered in the International Prospective Register of Systematic Reviews (PROSPERO)	32 European countries, (28 EU member states and the four EFTA countries (Iceland, Liechtenstein, Norway, and Switzerland)	×	/	/	Núñez-Núñez et al. [129]. https://doi.org/10.1016/j.cmi.2017.07.014 (accessed on 29 June 2025)
European Sales and Use of Antimicrobials for Veterinary Medicine (ESUAvet) annual surveillance reports	Europe	/	/	×	European Sales and Use of Antimicrobials for veterinary medicine (ESUAvet) [130]. https://www.ema.europa.eu/en/documents/report/european-sales-use-antimicrobials-veterinary-medicine-annual-surveillance-report-2023_en.pdf (accessed on 29 June 2025)
The Central Asian and Eastern European Surveillance of Antimicrobial Resistance (CAESAR) network—a joint initiative of the WHO Regional Office for Europe	30 countries of the EU and EEA	×	/	/	European Centre for Disease Prevention and Control and WHO Regional Office for Europe [127]. https://www.ecdc.europa.eu/sites/default/files/documents/antimicrobial-resistance-ECDC-WHO-executive-summary-2023-data.pdf (accessed on 29 June 2025)
EFSA (European Food Safety Authority) and ECDC (European Centre for Disease Prevention and Control). (2024).	27 EU Member States (MSs), the United Kingdom (Northern Ireland), and four non-MSs.	×	×	×	EFSA and ECDC [131,132]. https://doi.org/10.2903/j.efsa.2024.8583 (accessed on 29 June 2025)
The National Antimicrobial Resistance Monitoring System (NARMS)	USA	×	×	×	FDA Requests Public Comments to Inform Development of National Antimicrobial Resistance Monitoring System (NARMS) Strategic Plan for 2026–2030 [133]. https://www.fda.gov/animal-veterinary/antimicrobial-resistance/national-antimicrobial-resistance-monitoring-system (accessed on 29 June 2025)
Canadian Integrated Program for Antimicrobial Resistance Surveillance (CIPARS)	Canada	×	×	×	CIPARS [134]. https://www.canada.ca/en/public-health/services/surveillance/canadian-integrated-program-antimicrobial-resistance-surveillance-cipars.html (accessed on 29 June 2025)
Food and Agriculture Organization Antimicrobial Resistance Action Plan (FAO AMR)	150 countries worldwide	/	×	×	FAO [135]. https://openknowledge.fao.org/server/api/core/bitstreams/dd6c0ba1-fd85-4a3e-b398-53b610c35318/content (accessed on 29 June 2025)

×—presence; /—absence.

The essence is to reduce the need for antibiotics in food animal production systems by improving animal health through biosecurity measures, e.g., disease prevention (introduction of effective vaccines) and good hygiene and management practices, on farms and in slaughterhouses. Future research needs should be based on knowledge gaps, such as providing comparable national data on the occurrence of antibiotic resistance in relevant bacteria from the environment, food animals, food products, and humans, including the use of different types of antibiotics in different categories of food animals; actively using surveillance data in epidemiological studies and risk assessment, including the evaluation of interventions; improving the understanding of the mechanisms of resistance development and transmission; and developing new antibiotics and alternative approaches to antibiotic therapy. A sustainable and harmonized local, regional, and international coordinated joint system of AMR surveillance and suppression by public health and veterinary services is imperative for effective prevention of AMR in FPA. Data collection on AMR must be conducted at national and international levels for the “One Health” approach to combating AMR to be efficient and effective [136,137].

The “One Health” approach requires that researchers easily disseminate what they have learned or discovered, allowing other researchers to build on their findings. This is enabled by the use of standardized methods for communicating and archiving data. Support for the “One Health” initiative has been strengthened by the advent of whole genome sequencing (WGS), which has established genomic DNA as a standard data type and increased resolution between isolates, dramatically changing the way data is stored, shared, and analyzed in human pathogen surveillance. Also, storing and sharing genomic pathogen data and surveillance analysis as “open data” has enabled a truly open vision for the surveillance of all global pathogens, as demonstrated by the success of the open model for foodborne pathogen surveillance in the USA, the UK, Australia, Mexico, and Canada. Progress has been made through data on stress response genes of pathogen isolates, which have recently become available in the NCBI Pathogen Detection Isolate Browser (NPDIB) [138,139]. Existing research in the NPDIB database has mainly been related to AMR. Li et al. (2019) [140] examined prominent AMR genes from clinical pathogens carrying these genes in six countries and studied the trend of AMR gene occurrence over time. Yang et al., 2020 [141] extended the research in eight countries to foodborne pathogens and studied important AMR genes and the increasing trends of their occurrence in foodborne pathogens. Hua et al. (2020) [142] further investigated by comparing AMR genes sampled in these two pathogen species in the USA, and found that AMR was first detected in foodborne pathogens and then in clinical pathogens, suggesting that AMR genes may have been first found in foodborne pathogens (mostly transmitted by FPA) and then transferred to clinical pathogens (mostly transmitted by humans). A logical next step is to further identify and investigate genes that coordinate stress response and AMR.

## 7. Conclusions

Significant increases and intensifications of AMR have been observed, indicating the imperative for urgent improvements in monitoring and surveillance. It is necessary to overcome a number of shortcomings, such as structural ones, related to the organization of efficient and effective laboratory work and insufficient coordination between stakeholders and decision-makers in human, animal, and food sectors, which all constitute limitations of AMR surveillance in Europe and around the world. In the future, it will be of utmost importance to encourage professional and scientific management of the use of antimicrobial agents and control strategies that should suppress the riskiest MDR, zoonotic, or FBD infections. It goes without saying that empirical antibiotic therapy must be optimized (while minimizing/avoiding its prolongation). As alternatives with short time horizons, it is necessary to develop new drugs and vaccines. It is imperative to reduce the medical and economic burden that AMR inevitably leads to. It is also necessary to draw attention to the significant heterogeneity in the results of human studies and FPA and the enigma surrounding the effectiveness of strategies that limit the use of antibiotics to limit the emergence of antibiotic resistance. The new initiatives listed in Table 1 (intervention strategies to address the global challenge associated with AMR) and Table 2 (AMR monitoring and surveillance programs) are valuable tools for improving existing oversight, with ongoing activities that would be coordinated at the global level with the participation of political elites, without procrastination. Even better coordination between all stakeholders is needed, with specific recommendations for policy changes or research priorities contributing to greater impact, such as proposing standardized global surveillance protocols or funding mechanisms for alternatives to antibiotics. It is necessary to permanently improve and expand the NPDIB database, and fortify it with the results of next steps to identify and investigate genes that coordinate stress response and AMR”.

## Figures and Tables

**Table 1:** Intervention strategies to address the global challenge associated with AMR.

	Strategy	Approach	Flaws (Disadvantages)	Strengths (Benefits)
1.	Antibiotic (ABs) management	Targeted use of ABs, therapeutic administration and dosage must be optimized, ethical, and traceable.	Changes in the habits of patients and healthcare professionals are necessary.	The strategy reduces the emergence of resistance, maintaining AB efficacy at a desirable, justified level.
2.	New generations of ABs	Research and development in the creation of new ABs to eliminate existing and prevent new bacterial mechanisms that lead to AMR.	It is expensive, the development and registration of new ABs is lengthy, and there is a potential for cross-resistance to occur.	Reduces or prevents the emergence of AMR to available ABs already in use.
3.	Combining AB therapies	Treatment of infections using multiple ABs with different mechanisms of action.	Dosage is complicated, risk of AB antagonism and side effects.	Potential synergistic effects increase efficiency and effectiveness and reduce the AMR.
4.	Probiotics and Prebiotics	Promoting the growth of beneficial bacteria to outcompete harmful strains.	Limited knowledge of optimal strains, challenges in colonization and persistence.	Supports healthy microbiota, reduces space for pathogens.
5.	Immunotherapy (Vaccination)	Developing and boosting the immune response to fight against infections.	Live attenuated vaccines carry the risk of returning virulence and pathogenicity. Killed vaccines are safe but create weaker immunity with the need for revaccination; they are often specific for certain infections, i.e., only for certain strains (genotypes) of the virus; potential for the development of autoimmune processes.	Effective against various types and strains of microorganisms, long-term protection is possible.
6.	Phage Therapy	Using bacteriophages (viruses that multiply extremely quickly in bacteria and lead to their disintegration) to target specific bacterial strains.	Insufficient knowledge about phage-bacterial interactions, regulatory issues.	Highly targeted perspective, can be expeditiously modified.
7.	Breeding animals with better immunity	Animals with better immunity are easier to gain, healthier, and more resistant to breeding or infectious diseases.	Complex and uncertain, long-term creation is primarily achieved by genetic engineering.	Lower mortality, improved animal health and welfare, lower costs of ABs purchasing and vet services
8.	Implementation of stocking density standards or regulations	Prevention of the occurrence of various diseases, especially respiratory ones, which require metaphylaxis or mass therapy of sick or suspected sick animals with antibiotics	Reduced capacity of facilities for housing animals.	Lower costs of ABs purchasing and vet services; healthier animals, less difficult occurrence of infections and/or transfer/spread of infectious agents (especially in respiratory infections).
9.	Changing the purpose of ABs available in use	Identifying non-AB agents with AM power.	Limited options and number of ABs, dose optimization is a reality, as are side effects.	Lower costs due to potentially faster development.
10.	Alternatives to ABs	Creating non-AB treatments (AM peptides or bacteriocins; Plant-based drugs);	Insufficient clinical data support; toxicity and delivery method are still challenging.	Lower risk of AMR occurrence, various mechanisms; natural AM agents are biodegradable, with low allergenic potential.
11.	Using nutritional (natural) supplements instead of ABs	Plant-Derived Substances (polyphenols, alkaloids, and tannins) present a great potential for use, like antimicrobials or as AB resistance modifiers.	May cause side effects, interfere with the effects of other supplements or medications; In inappropriate, high doses, they can be toxic.	Do not normally cause AMR; Different mechanisms of action that can overcome AMR and the reduced side effects.
12.	Knowledge transfer and education, creating a positive public opinion for solving the AMR issue	Promoting good hygiene practice, knowledge of AB use, and understanding of AMR.	Reduces unnecessary and improper AB demand and misuse.	Continued efforts are expected and required: the change in awareness of the problems associated with AMR is gradual, and the impact is difficult to measure and assess.
13.	Surveillance Systems	Monitoring and tracking resistance patterns to inform treatment guidelines.	Resource-intensive, challenges in data sharing and harmonization.	Provides real-time data, guides treatment decisions.
14.	Artificial intelligence (AI) and applications in precision agriculture.	Optimizing AB use in agriculture; early disease detection and diagnosis; preventing AMR with predictions; precise application of treatments in agriculture; improved animal welfare; developing new AM drugs; amplifying surveillance and monitoring.	Insufficient farmer trust, higher investment required, possible only in industrial food production.	Quality and Availability of (Big) Data; Model Explainability; Ethical issues; Fusing with Existing Systems.
15.	Environmental Regulations	Limiting or reducing AB use in agronomy and the food industry to limit AMR transfer.	Regulatory enforcement, global coordination, and economic implications.	Mitigates selection pressure for resistance.
16.	One Health Approach	Coordinating efforts across human, animal, and environmental health to tackle resistance.	A multi-/interdisciplinary approach is necessary for collaboration, challenges in communication and policy alignment.	Addresses complex sources of AMR spread.

## Data Availability

No new data were created or analyzed in this study. Data sharing is not applicable to this article.
